# Supplementary material for: Triggering MSR1 promotes JNK‐mediated inflammation in IL‐4‐activated macrophages
Source: EMBO J. 2019 Apr 26;38(11):e100299. doi: 10.15252/embj.2018100299 (PMC6545745; doi:10.15252/embj.2018100299)
Supplement: Supplementary file 1 — Appendix [file EMBJ-38-e100299-s001.pdf]

Table of Content:

Appendix Table S1: Ubiquitylated proteins and their intensities identified in Halo-TAB2 TUBE pull-downs from phagosomes of M2(IL-4) macrophages.

Appendix Figure S1: Validation of IL4-activation.

Appendix Figure S2: Validation of phagosome proteomics data and specific regulated phagosome protein complexes.

Appendix Figure S3: Immunofluorescence of TAB1, TAB2 and MKK7 on M0 and M2 phagosomes.

Appendix Figure S4: Immunofluorescence of TAB1, TAB2 and MKK7 on M0 and M2 phagosomes.

Appendix Figure S5: Identification of MSR1 expression and K63 polyubiquitylation on phagosomes in activated macrophages.

Appendix Figure S6: oxLDL induces a MSR1-dependent pro-inflammatory switch and JNK inhibition reverses MSR1-dependent pro-inflammatory activation.

Appendix Figure S7: Quantitation of flow cytometry data in Figure EV6A.

Appendix Figure S8: Triggering of MSR1 does not seem to activate NF-κB pathway.

Appendix Table S1. Ubiquitylated proteins and their intensities identified in Halo-TAB2 TUBE pull-downs from phagosomes of M2(IL-4) macrophages

**Gene name**	**Protein**	**Intensity TAB2-mut**	**Intensity TAB2**	**Ubiquitylated position**
Ubc	Polyubiquitin-C	2.7×10^{4}	3.9×10^{5}	63;11;48;1;29;6;27;33
**Msr1**	**Macrophage scavenger receptor 1**	9.7×10^{4}	1.3×10^{7}	**27**
Slc43a2	Large neutral amino acids transporter small subunit 4	2.2×10^{6}	1.2×10^{7}	283;293;402;557
Ifitm3	Interferon-induced transmembrane protein 3	6.8×10^{4}	3.9×10^{6}	24
Rnf13	E3 ubiquitin-protein ligase RNF13	1.3×10^{5}	7.7×10^{6}	233
Csf1r	Macrophage colony-stimulating factor 1 receptor	3.3×10^{6}	8.0×10^{7}	572;810;625;584
C5ar1	Complement component 5a receptor 1	5.0×10^{6}	1.8×10^{7}	334
Slc38a2	Sodium-coupled neutral amino acid transporter 2	3.5×10^{5}	5.0×10^{6}	38;33
Serinc3	Serine incorporator 3	8.6×10^{4}	5.0×10^{5}	363
Slc7a2	Isoform 2 of Low affinity cationic amino acid transporter 2	0	3.5×10^{6}	449;632;654
Tyrobp	TYRO protein tyrosine kinase-binding protein	0	5.4×10^{5}	82
Lyn	Tyrosine-protein kinase Lyn	8.6×10^{6}	2.0×10^{7}	20
Unc93b1	Protein unc-93 homolog B1	4.1×10^{6}	1.4×10^{7}	197;582
Itm2b	Integral membrane protein 2B	1.6×10^{6}	1.1×10^{7}	13
Slc6a6	Sodium- and chloride-dependent taurine transporter	6.7×10^{5}	3.1×10^{7}	611;15;37
Lat2	Linker for activation of T-cells family member 2	0	1.0×10^{6}	39;84
Tfrc	Transferrin receptor protein 1	7.8×10^{6}	2.2×10^{7}	53;39
Abcg1	ATP-binding cassette sub-family G member 1	1.3×10^{6}	9.9×10^{6}	55
Slc40a1	Solute carrier family 40 member 1	1.9×10^{4}	2.2×10^{5}	269
Itch	E3 ubiquitin-protein ligase Itchy	1.9×10^{5}	8.1×10^{6}	192
Piezo1	Piezo-type mechanosensitive ion channel component 1	3.9×10^{4}	5.3×10^{6}	1914
Ednrb	Endothelin B receptor	3.4×10^{6}	9.5×10^{6}	417
Vamp8	Vesicle-associated membrane protein 8	1.0×10^{5}	2.7×10^{6}	47
Mgl2	Macrophage galactose N-acetyl-galactosamine	1.0×10^{7}	8.7×10^{7}	16
Hspa8	Heat shock 70 kDa protein 8	1.3×10^{7}	6.1×10^{7}	524
Slc23a2	Solute carrier family 23 member 2	7.9×10^{4}	3.2×10^{6}	11
Evi2a	Protein EVI2A	0	1.1×10^{6}	192;221
Itfg3	Protein ITFG3	5.8×10^{4}	1.8×10^{6}	36;38
Emr1	EGF-like module-containing mucin-like hormone receptor-like 1	3.6×10^{6}	1.9×10^{7}	743
Slc12a4	Solute carrier family 12 member 4	8.4×10^{4}	4.3×10^{6}	988
Slc3a2	4F2 cell-surface antigen heavy chain	3.5×10^{6}	3.2×10^{7}	42
Ttyh3	Protein tweety homolog 3	0	1.3×10^{7}	508
Clec4a3	C-type lectin domain family 4 member a3	0	1.6×10^{5}	13
Colec12	Collectin-12	0	2.3×10^{5}	2;17
Ms4a6d	Membrane-spanning 4-domains subfamily A member 6D	0	2.4×10^{5}	174
Slc5a3	Sodium/myo-inositol cotransporter	1.6×10^{5}	1.7×10^{6}	596
Trpv2	Transient receptor potential cation channel subfamily V member 2	3.5×10^{6}	2.3×10^{7}	51
Slc43a3	Solute carrier family 43 member 3	0	2.6×10^{5}	244

Appendix Figure Legends

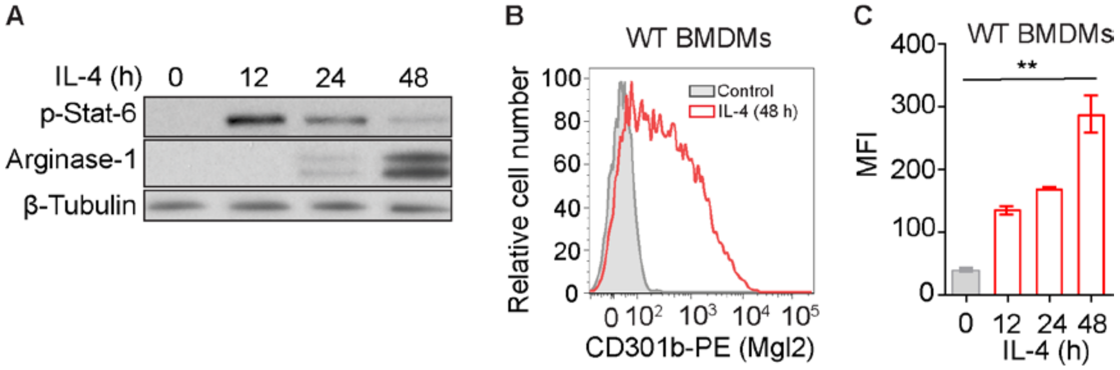

Appendix Figure S1: Validation of IL4-activation.

(A) Immunoblot analysis (IB) of IL-4- induced M2 markers including pStat-6 and Arginase-1. Macrophages were fully activated after 48 h as shown by strong induction of Arginase-1. Tubulin serves as loading control. (B) The cell surface levels of CD301b/Mgl2 in response to IL-4 stimulation. (C) Quantification of mean fluorescence intensity (MFI) of cell surface levels of CD301b/Mgl2 in response to IL-4 stimulation.

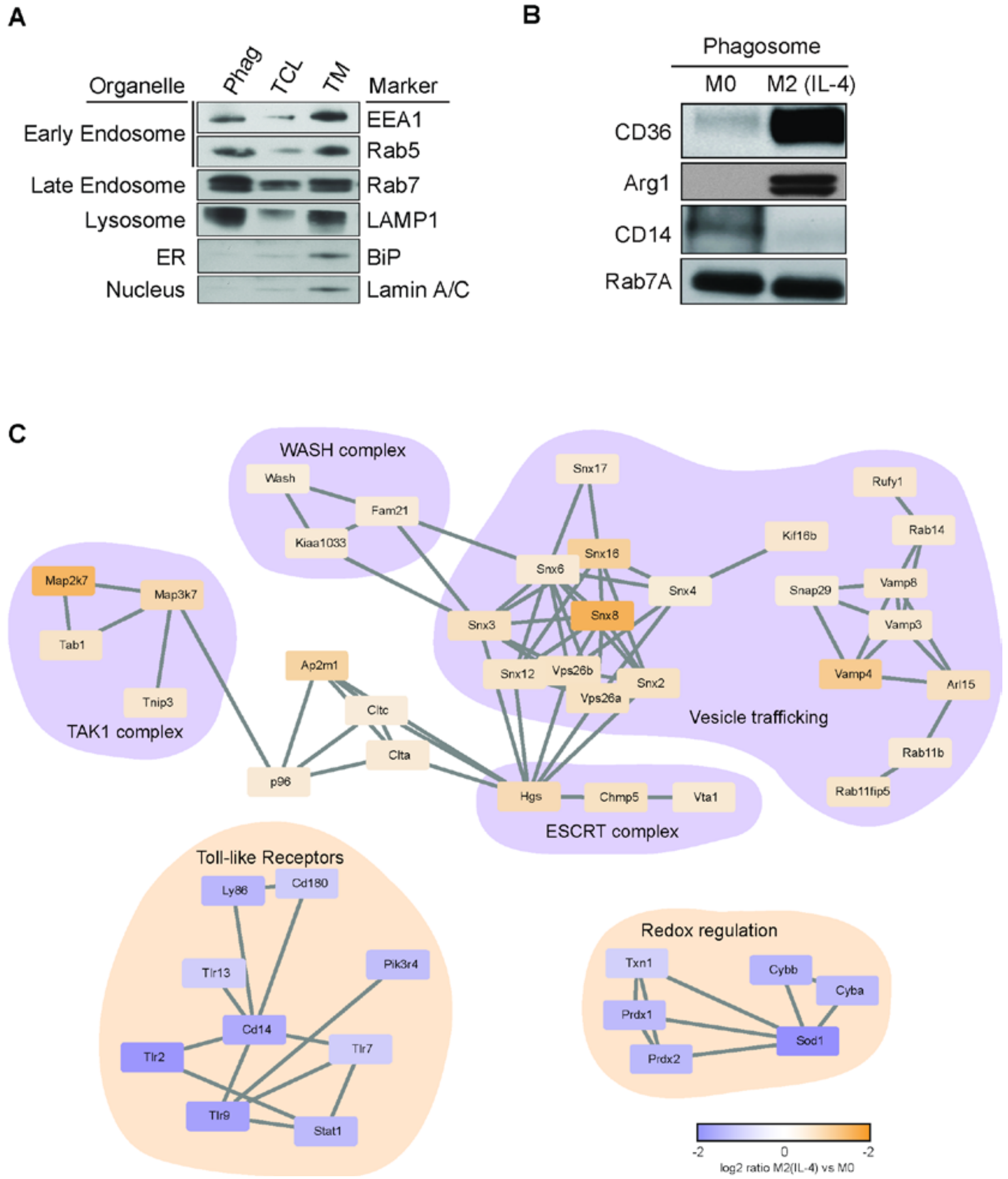

Appendix Figure S2: Validation of phagosome proteomics data and specific regulated phagosome protein complexes.

(A) IB of markers of early and late endosomes (EEA1, Rab5a and enriched on phagosomes, while nuclear and ER markers (Lamin A/C and BiP) are more abundant in total cell lysates (TCL) and total membrane (TM), respectively. (B) IB analysis validating selected proteins changing in proteomics data. (C) Protein–protein interaction network of phagosome proteome using the STRING database v10 (Szklarczyk D, et al, Nucleic Acid Res, 2015). Subnetworks positively affected by phagocytosis in M2(IL-4) macrophages were vesicular trafficking and signalling, while proteins associated with innate immune response and ROS were down-regulated. Proteins involved in vesicular trafficking and signalling, Redox regulation and Toll like receptor complexes are displayed. (A-B) Representative blots of two biological replicates.

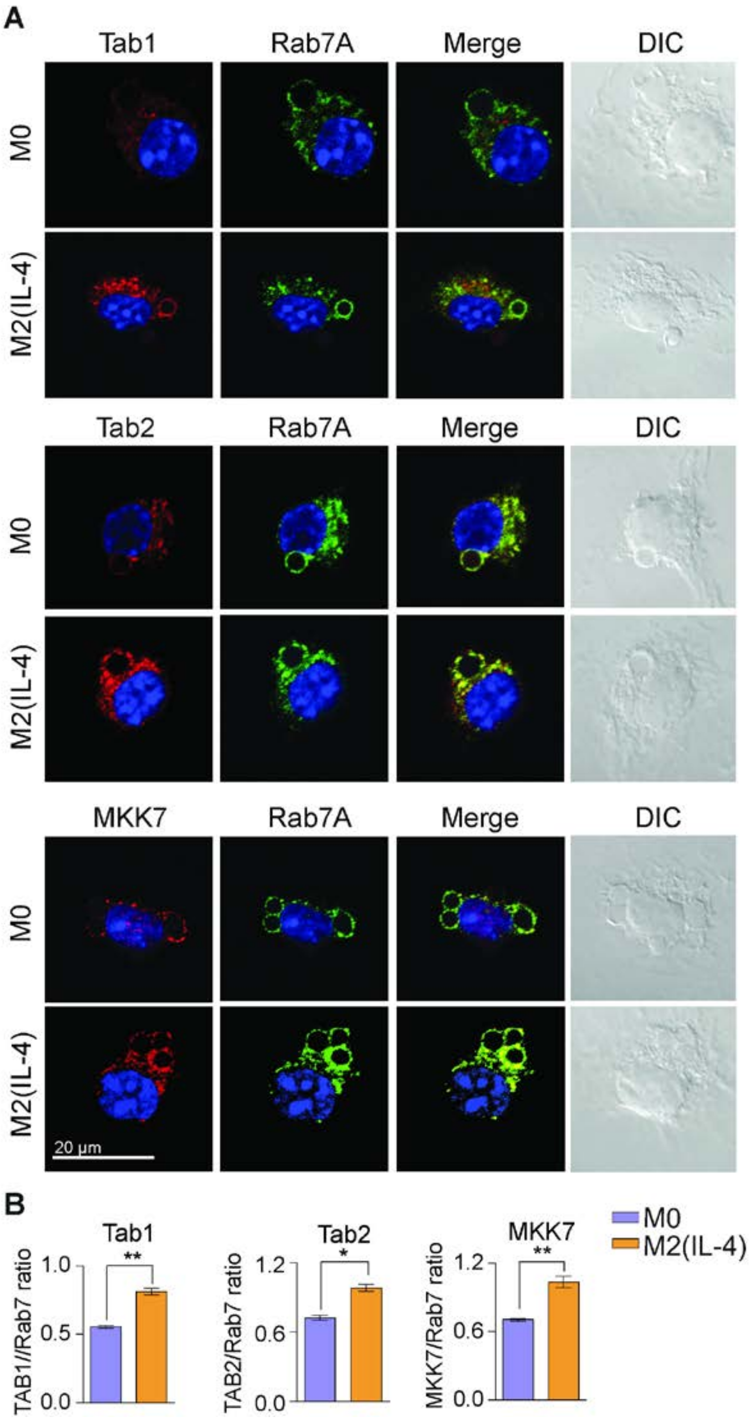

Appendix Figure S3: Immunofluorescence of TAB1, TAB2 and MKK7 on M0 and M2 phagosomes.

(A) Immunofluorescence micrographs showing co-localisation of TAB1, TAB2 and MKK7 proteins with Rab7a, a phagosomal marker, around 30 min phagosomes in M0 and M2(IL-4) macrophages. (B) Corresponding quantification of co-localisation of TAB1, TAB2 and MKK7 with Rab7a in M0 and M2(IL-4) macrophage phagosomes, plotted as a ratio of the fluorescence of the target protein to Rab7a fluorescence localized to phagosomes. Scale bar is 20 µm. Data are shown as mean ± SEM from three independent experiments. $^{**}p < 0.01$, $^{***}p < 0.001$; (Student's T-test).

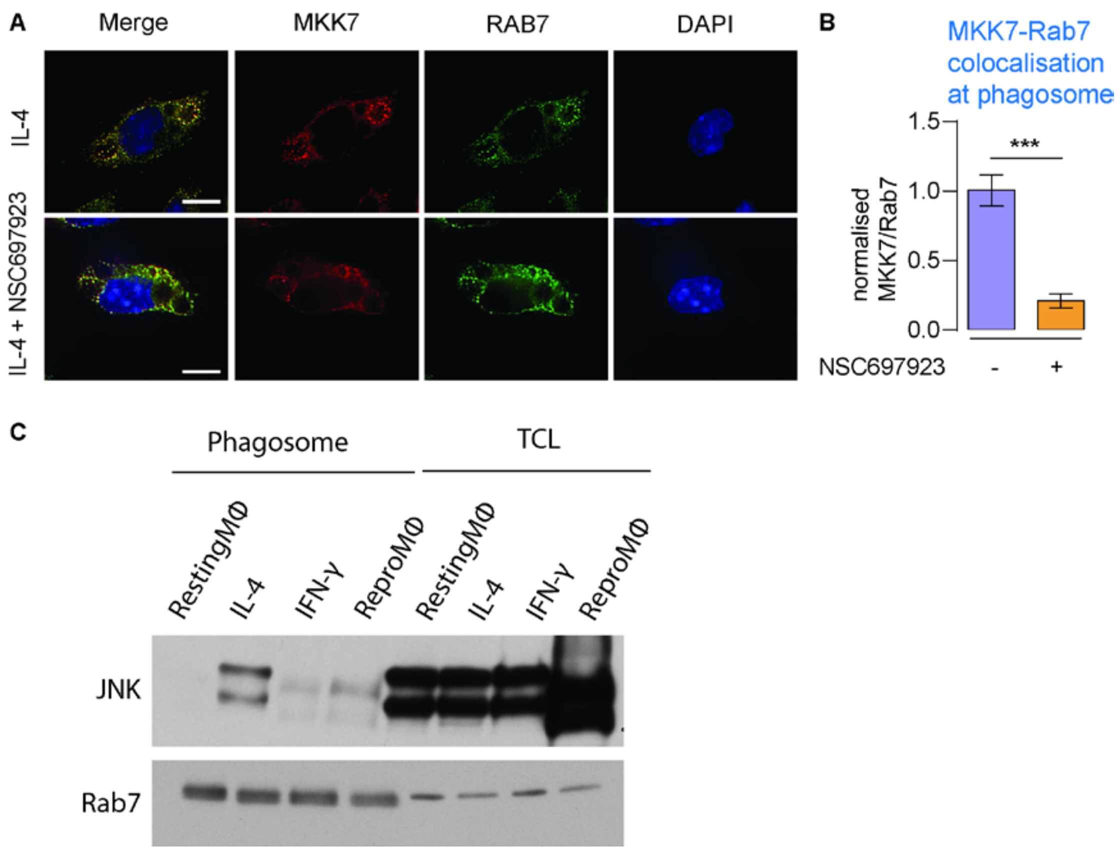

Appendix Figure S4: Immunofluorescence of TAB1, TAB2 and MKK7 on M0 and M2 phagosomes.

(A) Immunofluorescence micrographs showing co-localisation of MKK7 protein with Rab7a, a phagosomal marker, around 30 min phagosomes in M2(IL-4) macrophages. Co-localisation is reduced upon treatment with the UBC13 inhibitor NSC697923. (B) Corresponding quantification of co-localisation of MKK7 with Rab7a in M2(IL-4) macrophage phagosomes ± treatment with NSC697923, plotted as a ratio of the fluorescence of MKK7 to Rab7a fluorescence localized to phagosomes. Scale bar is 10 µm. Data are shown as mean ± SEM from three independent experiments. **p < 0.01, ***p < 0.001; (Student's T-test). (C) Full blot of JNK from Figure 3A. Phagosomes and total cell lysates (TCL) were isolate from resting, IL-4 activated (48 hrs), IFN-γ (24 hrs) and "reprogrammed" macrophages (24 hrs IL-4 followed by 24 hrs of IFN-γ). Data shows that IL-4 activation enhances specifically recruitment of JNK to the phagosome. Rab7 serves as loading control.

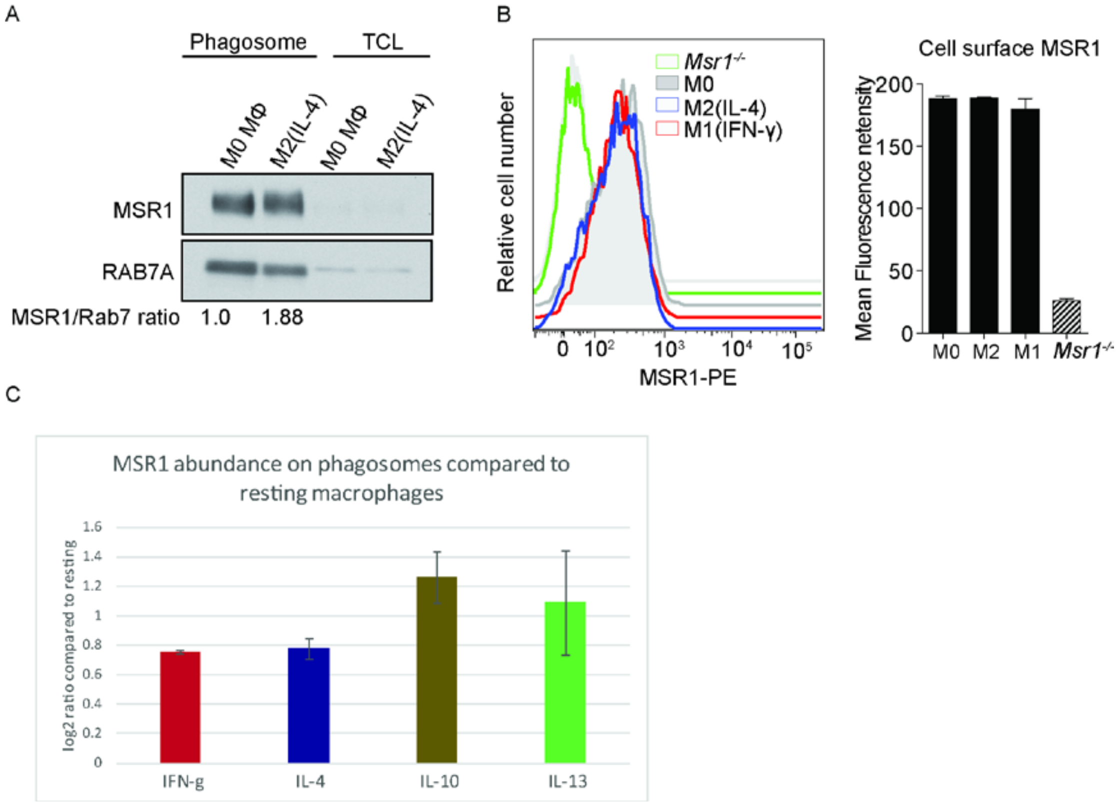

Appendix Figure S5: Identification of MSR1 expression and K63 polyubiquitylation on phagosomes in activated macrophages.

(A) Immunoblot of MSR1 showing light enrichment of the non-ubiquitylated form of the protein on phagosomes in M2(IL-4) macrophages compared to M0 macrophages. (B) Activation does not affect MSR1 surface expression. Flow cytometry analysis and corresponding quantification shows equal cell surface expression of MSR1 in M0, M2(IL-4) and M1(IFN-γ) macrophages. Data are shown as mean ± SEM from three independent experiments. MFI (Median Fluorescence Intensity). (C) Proteomics protein abundance data for MSR1 on 30 min old phagosomes from IFN-γ (100 U/ml, 24 hrs), IL-4 (20 ng/ml; 48 hrs), IL-10 (10 ng/ml; 48 hrs) and IL-13 (20 ng/ml; 48 hrs) activated macrophages compared to phagosomes from untreated/resting macrophages. The data shows that MSR1 is induced on phagosomes upon all of these treatments. Data are shown as mean ±standard deviation from three independent biological experiments.

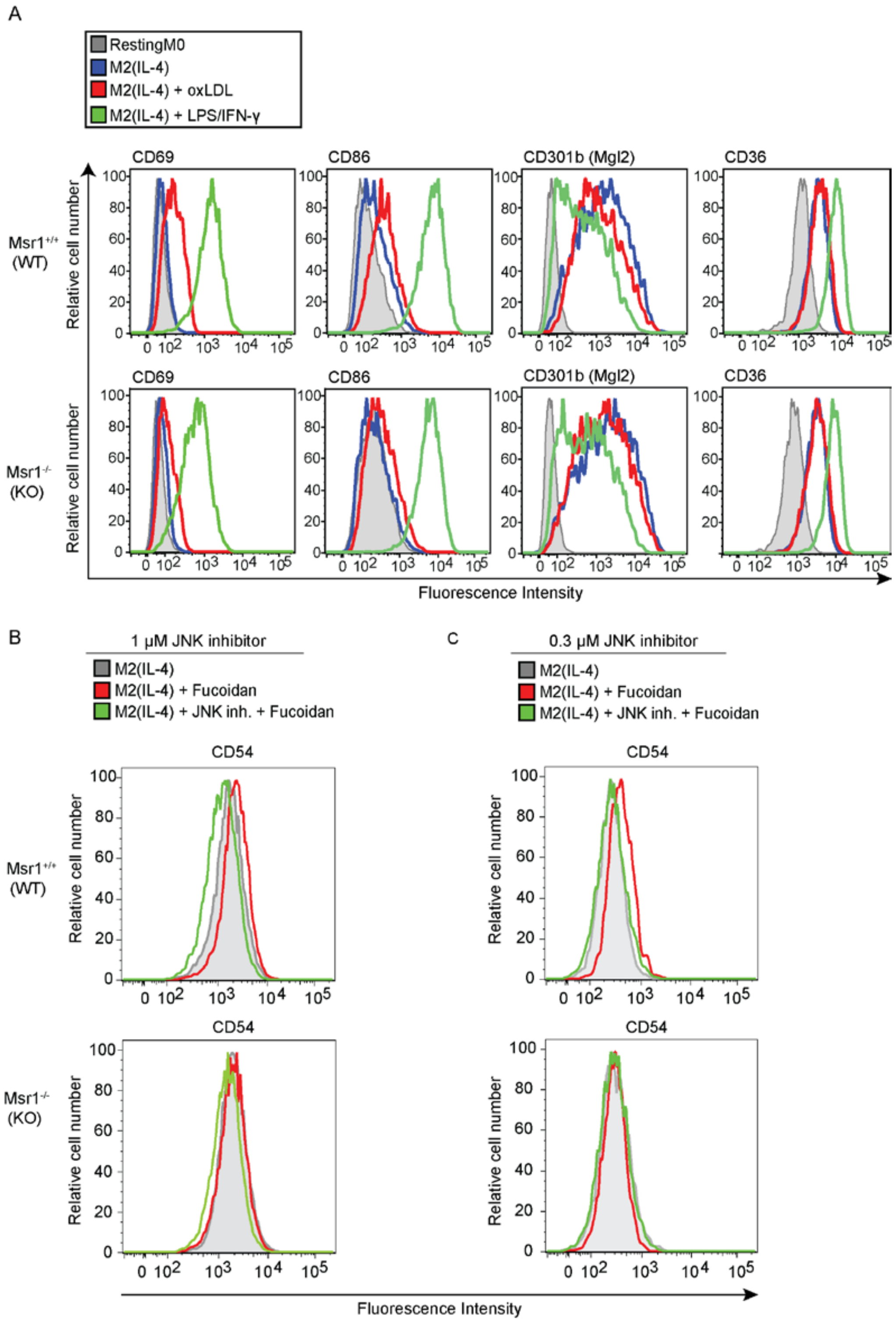

Appendix Figure S6: oxLDL induces a MSR1-dependent pro-inflammatory switch and JNK inhibition reverses MSR1-dependent pro-inflammatory activation.

(A) Flow cytometry analysis of cell surface expression of CD69, CD86, CD301b and CD36 of M2(IL-4) followed or not by either oxLDL or LPS/IFN-γ treatment. (B-C) Inhibition of JNK by 1 and 0.3 µM (C) of JNK-IN8 reverses the increase of proinflammatory activation of M2(IL-4) macrophages upon fucoidan treatment. Data representative of two independent experiments.

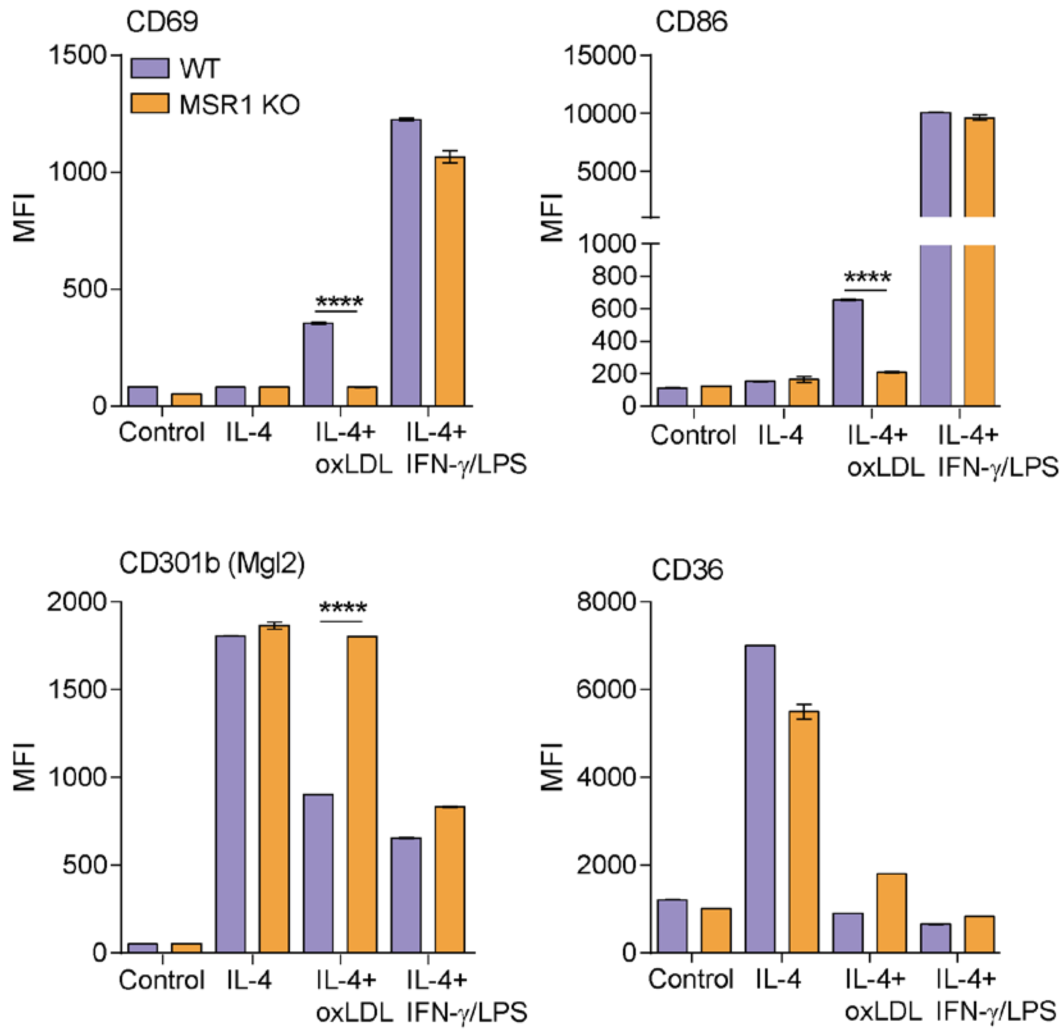

Appendix Figure S7: Quantitation of flow cytometry data in Appendix Figure S6A. Data shows that triggering of MSR1 through oxLDL induces a more pro-inflammatory phenotype in IL-4 activated WT BMDMs but not in MSR1 KO macrophages. Data are shown as mean ± SEM from three independent experiments. MFI (Median Fluorescence Intensity).

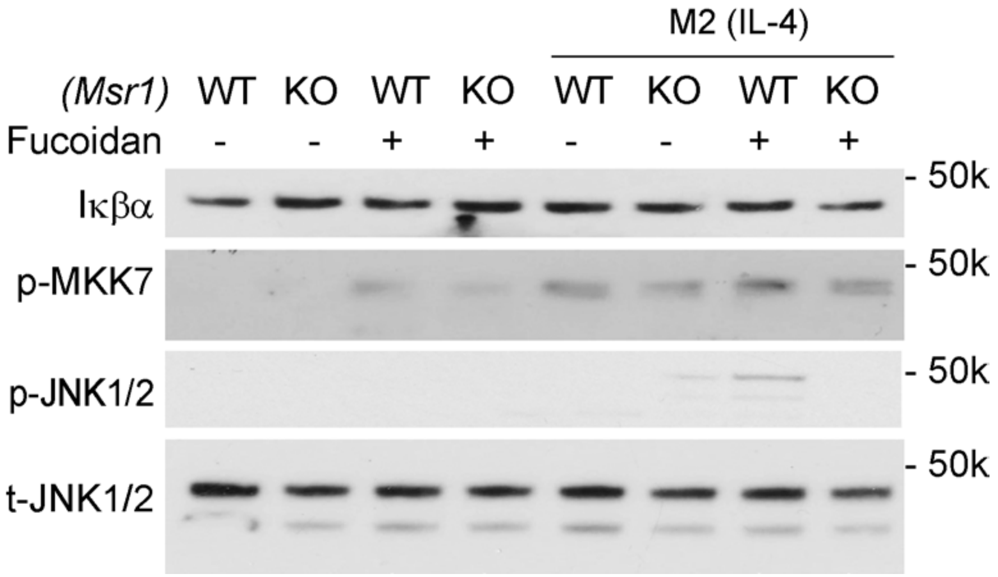

Appendix Figure S8: Triggering of MSR1 does not seem to activate NF-κB pathway. Immunoblot data shows that IκBα is not degraded in response to triggering of MSR1 with 50 μg/ml Fucoidan, indicating that the NF-κB pathway is not activated.
